# Increased Whole Blood Viscosity Is Associated with the Presence of Digital Ulcers in Systemic Sclerosis: Results from a Cross-Sectional Pilot Study

**DOI:** 10.1155/2017/3529214

**Published:** 2017-11-29

**Authors:** Peter Korsten, Timothy B. Niewold, Michael Zeisberg, Tammy O. Utset, Daniel Cho, Lawrence S. Zachary, Nadera J. Sweiss, Suncica Volkov

**Affiliations:** ^1^Department of Nephrology and Rheumatology, University Medical Center Goettingen, Goettingen, Germany; ^2^Division of Rheumatology and Department of Immunology, Mayo Clinic, Rochester, MN, USA; ^3^Department of Rheumatology, University of Chicago, Chicago, IL, USA; ^4^Rheovector LLC, King of Prussia, PA, USA; ^5^Section of Plastic and Reconstructive Surgery, Department of Surgery, University of Chicago, Chicago, IL, USA; ^6^Division of Rheumatology, University of Illinois at Chicago, Chicago, IL, USA; ^7^Division of Pulmonary, Critical Care, Sleep and Allergy, University of Illinois at Chicago, Chicago, IL, USA

## Abstract

**Objective:**

To investigate the role of whole blood viscosity in digital ulcer (DU) development in patients with diffuse and limited Systemic sclerosis.

**Methods:**

A convenience sample of patients with Systemic sclerosis (SSc) was selected from the adult Rheumatology clinic at the University of Chicago. The study group consisted of patients with SSc (with ulcers present, a history of ulcers, and no ulcers); the control group consisted of matched healthy Rheumatology clinic staff. WBV was measured using a scanning capillary viscometer at different shear rates (1–1000 1/s).

**Results:**

Whole blood viscosity as measured by a scanning capillary viscometer was increased in patients with SSc compared to healthy controls (*p* < 0.0001). Additionally, patients with present DU had significantly higher whole blood viscosity when compared to patients with a history of DU and patients with no history of DU (*p* < 0.0001). These findings were most pronounced at lower shear rates between 1 and 10 1/s.

**Conclusion:**

Whole blood viscosity might be a contributing factor in DU development in patients with SSc. Further studies with larger patient cohorts are required to fully evaluate how increased WBV contributes to the development of DU and whether the currently available treatment options improve the microcirculation by influencing WBV.

## 1. Introduction

Systemic sclerosis (SSc) is an autoimmune connective tissue disease characterized by cutaneous and visceral fibrosis, but also diffuse vascular pathology [1]. A majority of SSc patients have Raynaud's phenomenon (RP), which is an exaggerated vascular response to stimuli, such as cold temperature or emotional stress. Up to 60% of SSc patients with RP develop digital ulcers (DU) in the course of their disease [2].

The pathogenesis of DU in SSc is incompletely understood. Obliterating vasculopathy and inflammatory mediators may play a role [1]. Changes of hemorheological properties (whole blood viscosity (WBV)) in patients with secondary RP (including SSc) were already reported previously [3]. However, few studies have further investigated these findings.

Elevated WBV has been recognized as a risk factor for adverse cardiovascular events in the general population, even after adjusting for traditional risk factors such as age, male gender, obesity, hypertension, diabetes, and smoking [4]. Also, cardiovascular events in patients with chronic kidney disease have been found to be associated with increased whole blood viscosity [5].

WBV is determined by hematocrit, plasma viscosity, red blood cell aggregation, and deformability. It is influenced by shear stress and other factors. Plasma viscosity is the intrinsic flow resistance of plasma and depends on plasma protein concentrations, predominantly fibrinogen.

Rotational viscometers have been the standard technique by which blood viscosity was measured for clinical studies [6]. Disadvantages of these systems are the inability to produce viscosity data at multiple shear rates and the need to treat blood samples with anticoagulation during viscosity testing. Anticoagulated blood inhibits cell-cell interactions altering (decreasing) WBV at low shear rates [6]. Plasma and serum viscosity as well as their components can be examined by rotational viscometers, but a more reliable and reproducible way to measure WBV would help to better understand what occurs in the vessel under different conditions in terms of shear rates.

The scanning capillary viscometer (SCV Rheolog™ by Rheovector, Exton, PA, USA) used in this study is able to quickly measure WBV at a wider range of shear rates. To examine WBV with the SCV, blood components do not have to be separated (in contrast to serum or plasma viscosity) and therefore reflect physiological conditions in the blood vessel more appropriately. Increased shear rates lead to a decrease in viscosity. In areas of low shear rates, such as the digits, WBV is therefore higher. If WBV increases at a constant systolic blood pressure, peripheral vascular resistance increases, thus reducing blood flow. This could lead to development of DU in SSc. When WBV decreases, circulation improves which results in increased perfusion, particularly in the microvasculature. A pathogenic role of WBV in DU development in SSc is therefore likely. The validity of the SCV to measure WBV has been established in several studies [7].

The present study is the first study using the SCV to investigate WBV at different shear rates in relation to the presence or absence of DU in patients with SSc and healthy controls.

## 2. Patients and Methods

### 2.1. Study Population

We prospectively included 33 patients with SSc and 13 matched control subjects between October 2005 and April 2006. All subjects were 18 years or older and SSc patients met the 2013 American College of Rheumatology (ACR)/European League Against Rheumatism classification criteria for SSc [8] as well as the 1980 ACR classification criteria, which were in use at the time of study conduction.

“Presence of digital ulcer” was defined as break in the skin on the fingertip, with or without granulation tissue, which had not completed the healing process. Patients were recruited during regular office visits to the adult Rheumatology clinic at the University of Chicago. Control subjects were recruited from healthy university personnel and were matched to the SSc group. The Institutional Review Board at the University of Chicago approved the study and all subjects gave written informed consent.

Epidemiological data on all 46 subjects was obtained by routine use of structured documentation; measurements of whole blood viscosity were performed in all patients. Disease activity was assessed by physical examination and digital ulcer assessment.

### 2.2. Capillary Viscometer

4 to 6 mL of blood with standard venipuncture were obtained and immediately processed. We used a scanning capillary viscometer (SCV, Rheovector LLC, Exton, PA) to measure WBV.

The SCV has a Food and Drug Administration Investigational Device Exempt status. It was developed in 2001 and has been validated to measure WBV (mPa × second) at incremental shear rates from 1 to 1000 seconds^−1^. The SCV is a portable device that maintains blood samples in a closed system. This system is coated with biocompatible material and is temperature controlled, simulating in vivo conditions. The blood is led into a U-formed capillary, filling one of the vertical columns higher than the other, before permitting the columns to equilibrate through gravity forces. The flow occurs rapidly at first, slowing down over time until the blood levels in the two tubes are nearly equal. While the blood is flowing, the system records the height of the two columns over a defined time period (3 minutes). The flow rate (determined by the rate of change in height of the columns of blood) is directly related to the pressure drop across the capillary tube, from which the shear rate, viscosity, and shear stress of the sample can be mathematically derived. The raw data are presented graphically and then numerically on an attached computer.

The determination of a full profile (from a shear rate of 1 to 1000 s^−1^) is important for the evaluation of blood properties, as variations of viscosity at low shear rates may be especially important in the pathophysiology of digital ulcer DU formation.

### 2.3. Statistical Analysis

All data were analyzed using GraphPad Prism version 6.0 for Mac OS X (GraphPad Software, La Jolla California USA, https://www.graphpad.com). Fisher's exact, Chi-square, Student's *t*-test, or Mann–Whitney test was used to compare the demographic characteristics between patients and controls as appropriate for parametric or nonparametric data.

WBV values were log-transformed and plotted against shear rates in *X*-*Y* plots. The data were analyzed using curve-fit methods, and a strong fit was achieved using a one-phase exponential decay curve to model the WBV data at various shear rates. WBV versus shear rate curves were compared between SSc patients and the control group as well as within SSc with ulcers versus SSc with a history of ulcers versus SSc without ulcers. To test for differences between the curves, an extra sum-of-squares *F* test was used, and *p* values shown on the graph are derived from this test. This tests the hypothesis that the data points from each condition (for ex. patients versus control) represent the same curve or a different curve. Low *p* values allow for the null hypothesis (same curve) to be rejected.

## 3. Results

### 3.1. Patients and Controls

The demographic characteristics of 33 SSc patients and 13 control subjects are presented in Table 1. There were 31 women and two men in the SSc group (10 African Americans, 20 Caucasians, and 3 Hispanics). The control group consisted of twelve women and one man (8 African Americans, 3 Caucasians, and 2 Hispanics). Mean age was 48.76 ± 12.66 for SSc patients and 41.15 ± 13.27 for controls. There were no significant differences in terms of age, gender, body mass index (BMI), and ethnicity between groups.

### 3.2. Characteristics of the Systemic Sclerosis Group

Type (diffuse or limited SSc), disease duration, and digital ulcer assessment of the Systemic sclerosis group were recorded (Table 1). 19 patients (57%) had diffuse SSc while 14 patients (43%) had limited SSc. Disease duration ranged from 1 to 29 years. Presence or absence of ulcers was assessed by history and physical examination. Four patients (12.1%) had DU at the time of the study, 17 had a history of DU (51.5%), and 12 (36.4%) had no prior history of DU (36%).

### 3.3. Comparison of Whole Blood Viscosity Measured at Different Shear Rates between SSc Patients and Control Group

**Figure 1:**
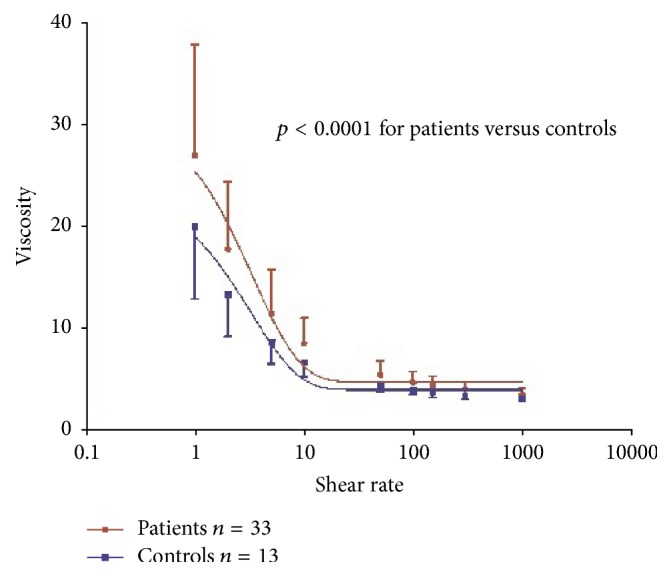
*Whole blood viscosity compared between patients (red) and controls (blue)*. WBV was significantly higher in the Systemic sclerosis group compared to healthy controls (*p* < 0.0001). This difference was most pronounced at shear rates between 1 and 10 s^−1^; however, shear rates were significantly different at all rates.

First, we compared WBV of the whole patient cohort with healthy control patients. Shear rates were measured ranging from 1 to 1000 s^−1^. Values were significantly higher in SSc patients versus controls (*p* < 0.0001). The absolute difference was greatest at low share rates (between 1 and 10 s^−1^) (Figure 1).

### 3.4. Comparison of Whole Blood Viscosity between Systemic Sclerosis Patients with Ulcers, a History of Ulcers, and a Negative History for Ulcers

**Figure 2:**
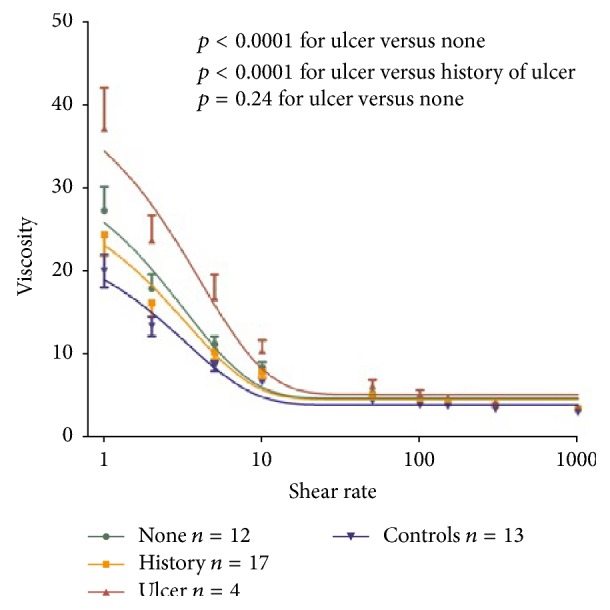
*Whole blood viscosity in Systemic sclerosis patients*. WBV was compared between patients with ulcers (red), a history of ulcers (yellow), and no history of ulcers (green). Control patients are also depicted (purple). WBV was significantly higher in patients with ulcers versus patients with a history of ulcers (*p* < 0.0001) and versus patients with no history of ulcers (*p* < 0.0001). By contrast, there was no difference between patients with a history of ulcers versus patients with no history of ulcers (*p* = 0.24).

Next, we investigated whether there were any detectable differences between patients with DU, a history of DU, and those with a negative history of DU (Figure 2). We found that WBV was highest for patients with ulcers compared to those with a history of DU and no history of DU (*p* < 0.0001). There was no difference between patients with a history of DU compared to patients with no history of DU (*p* = 0.24), suggesting that an increase in WBV is pathophysiologically important in DU formation.

## 4. Discussion

The present study is the first examining WBV related to DU in patients with SSc using a SCTV. There are several important limitations of our study: First, the number of individuals studied was small. Second, only four patients had ulcers at the time of the examination. Also, we did not follow the patients for a longer period, which is why we cannot make any recommendations whether an increase in WBV from baseline in an individual patient translates into a higher risk for the development of DU. Despite these limitations, our study is the first to show that WBV might play a critical role in the development of active DU in patients with SSc.

The concept of altered hemorheological variables in various disease states has been studied for over 40 years. An early study by Goyle and Dormandy investigated patients with primary RP versus controls and found an increase in blood viscosity in persons with RP [9]. Nevertheless, other investigators postulated that blood viscosity is different between patients with primary RP and secondary RP [10]. Of note, these studies used different methods of WBV measurements and findings may not directly apply to our cohort. The primary purpose of this study was to analyze WBV in relation to presence or absence of DU in SSc patients, which is why we did not include other control groups. In our experience, persons with primary RP show no statistically different WBV measurements compared to healthy controls (data not shown).

Based on the results of our pilot study, studies investigating WBV in Systemic sclerosis should be performed prospectively over a longer time to assess patients at different time points during the course of their disease. Our study was a cross-sectional study intended as a hypothesis-generating study. We therefore did not make any measurements of WBV after ulcers have healed, which is another limitation. We would nevertheless suppose that WBV would be comparable to levels observed in the group with a history of ulcers, which served as comparator within the SSc group (see Figure 2).

It is hypothesized that immune-mediated inflammation in connective tissue diseases may initiate or promote atherosclerosis, which may partially explain higher incidence of cardiovascular events in this group of patients. Acute phase reactants, such as fibrinogen or increased levels of immunoglobulins, influence whole blood viscosity in autoimmune diseases, such as rheumatoid arthritis (RA) or systemic lupus erythematosus (SLE) [11]. Patients with SLE, for example, who have increased WBV, were found to have a higher incidence of arterial thromboembolic events [12]. In rheumatoid arthritis, citrullinated fibrinogen might contribute to the observed increase in WBV and has been implicated in special situations such as Felty's syndrome (reviewed by [11]). The concept of alterations in blood rheology is therefore not specific for Systemic sclerosis. Investigations of blood rheology have gained interest in cardiovascular diseases. In the recent studies by Peters et al. [4] and Celik et al. [5], WBV was measured at higher share rates and by slightly different methods because these were macrovascular studies. In these studies, higher WBV correlated with the incidence of cardiovascular events. These findings show that an increase in WBV is not specific for any disease but rather reflects a distinct pathology in a given situation.

The main finding of our study, although it is based on a low number of subjects, is that WBV is increased in SSc compared with healthy controls and was also different within the SSc group depending on the presence or absence of DU. These latter findings may have some implications. Currently, standard treatments for RP/DU in SSc include vasodilatory substances, such as calcium antagonists or, in more severe cases, iloprost infusion therapy. Bosentan, an endothelin-receptor antagonist, is recommended for the prevention of new ulcers in patients who have had DU [13]. After the results of the RAPIDS-1 and RAPIDS-2 and SEDUCE trials [14–16], the main focus in treatment and prevention of critical or refractory DU therefore relies on endothelin-1 antagonists or phosphodiesterase inhibitors.

Taken together, evidence-based treatment options are quite limited for this manifestation. It may be interesting to incorporate measurements of blood rheology in future treatment studies on RP/DU in SSc patients since not every medication might exert the same effect on blood viscosity. It is clear that other treatments are required since not all patients respond to standard treatment.

Earlier studies by Jacobs et al. [17] found that plasma viscosity was significantly lower in patients with RP after plasmapheresis and was associated with enhanced skin capillary blood flow, improvement in symptoms, and healing of the skin ulcers. Overall, plasma exchange in RP or DU has been investigated in only few studies despite seemingly promising results [3, 17, 18] and is often not mentioned in the current literature [19]. Even though therapeutic apheresis is invasive, in our experience, it is a safe and well tolerated procedure with few side effects or complications and it might be considered as a therapeutic option in refractory ulcers. While we cannot draw any conclusions on the effectiveness of plasmapheresis in refractory DU based on our data, it may be an option in recalcitrant cases.

In conclusion, despite the aforementioned limitations, our study shows that WBV is increased in SSc patients versus healthy controls and also in patients with active DU. Future studies of WBV in SSc should aim to follow a larger cohort of patients for a longer period of time to measure WBV at times of active DU and after healing and also to assess treatment effects on blood viscosity.

## Figures and Tables

**Table 1:** Characteristics of the studied patients.

	Systemic sclerosis (*n* = 33)	Controls (*n* = 13)	*p* value
Gender	31 women (94%) 2 men (6%)	12 women (92%) 1 man (8%)	1.0

Race	20 Caucasians (60.6%) 10 African Americans (30.3%) 3 Hispanics (9.1%)	8 Caucasians (61.5%) 3 African Americans (23.1%) 2 Hispanics (15.4%)	0.9309

Age range	48.76 ± 12.66	41.15 ± 13.27	0.0772

Disease type	Diffuse SSc (*n* = 19, 57%) Limited SSc (*n* = 14, 43%)	—	

Disease duration	1–29 years	—	

Digital ulcers	Present ulcers (*n* = 4, 12.1%) History of ulcers (*n* = 17, 51.5%) No history of ulcers (*n* = 12, 36.4%)	—	

BMI: body mass index, CRP: C-reactive protein, and SSc: Systemic sclerosis.
